# Secular trends in the incidence and survival of all leukemia types in the United States from 1975 to 2017

**DOI:** 10.7150/jca.52186

**Published:** 2021-02-22

**Authors:** Xiaorong Yang, Hui Chen, Jinyu Man, Tongchao Zhang, Xiaolin Yin, Qiufeng He, Ming Lu

**Affiliations:** 1 Clinical Epidemiology Unit, Qilu Hospital of Shandong University, Jinan, China.; 2 Clinical Research Center of Shandong University, Qilu Hospital, Cheeloo College of Medicine, Shandong University, Jinan, China.; 3 Department of Epidemiology and Health Statistics, School of Public Health, Cheeloo College of Medicine, Shandong University, Jinan, China.

**Keywords:** leukemia, incidence, survival, secular trend, SEER

## Abstract

**Background:** Various studies have indicated that the prognosis of leukemia has been improved in recent years, but the secular trends of incidence and long-term survival of all leukemia have not been thoroughly examined.

**Methods:** We estimated the leukemia incidence and 5-year survival rate along with the temporal trends by sex, race, age, and subtype in the United States over the past four decades using Surveillance, Epidemiology, and End Results (SEER) database.

**Results:** The overall incidence of leukemia steadily increased from 12.39/100 000 in 1975 to 14.65/100 000 in 2011, and then began to decline in recent years (13.73/100 000 in 2017), with average annual percent changes (APC) of 0.350 (*P*<0.001). The 5-year relative survival rate of leukemia patients significantly improved from 33.2% in 1975 to 66.1% in 2012 (APC=1.980, *P*<0.001). The main subtypes of leukemia, including acute lymphoblastic leukemia, acute myeloid leukemia, chronic lymphocytic leukemia, and chronic myeloid leukemia, increased in most age groups; conversely, the incidences of all other subtypes were gradually declined during the monitoring period. The incremental advancement in leukemia prognosis had been achieved in almost all histological subtypes, especially among young patients.

**Conclusions:** Based on SEER data, the leukemia incidence increased gradually over the past decades, and then began to decline in recent years in the United States. The 5-year relative survival rate increased incrementally over time, especially among young patients. However, the huge disparities among different sexes, races, histological subtypes, and age groups, emphasize that precise causes control and innovative treatments need to be developed to reduce the incidence and improve the prognosis, especially among specific populations.

## Introduction

Leukemia is a group of cancers derived from the body's hematopoietic tissues, including the bone marrow and the lymphatic system. The GLOBOCAN estimated that about 410 000 new leukemia cases and 310 000 leukemia-related deaths occurred in the world in 2018 1. The subtypes of leukemia showed distinct characteristics in people of different ages: acute lymphoblastic leukemia (ALL) has long been the most common cause of cancer-related deaths under 15 years 2, 3, in contrast, acute myeloid leukemia (AML) usually occurred in the elderly, with a median age of 67 years and around 30% of AML patients over 75 years 4. In addition, higher leukemia incidence in children can be observed in developed countries, compared with developing countries 5, 6. The heterogeneity of leukemia of different sexes, races, ages, and subtypes has been reported, however, the corresponding secular trend of leukemia incidence in recent decades is still unclear.

With the progress of hematopoietic stem cell transplantation and new chemotherapy treatments, the survival rate of most leukemia patients has greatly improved over the past decades. For example, the 5-year survival rate for ALL patients increased from 35% in the mid-1970s to 70% in 2009 7. However, the disparities should be noted: although the 5-year survival rate archives 90% in ALL pediatric patients, only 25% of ALL patients over 50 years old can survive for 5 years 8, 9. Moreover, the incidence of ALL in the Black population was lower than the White population in the United States, however, the inferior survival could be observed in the Black population 3, 10. The prognosis of AML children remains relatively worse than ALL children, with a 5-year survival rate of 66% and 90%, respectively 3.

Accumulating evidence has indicated that the incidence and long-term survival rate of leukemia are characterized by its heterogeneity among different sexes, races, ages, and histological subtypes; however, these published researches usually focus on one subtype of leukemia or in a specific population, such as children or the elderly 5, 8, 9, 11-13. Only Xie et al. evaluated the change of incidence and survival of overall leukemia in the United States from 1973 to 1998 14, but the updated progress was imperative. Recently, Bispo et al. reported the latest incidence and survival rates of leukemia and lymphoma by subtype, age, and race, but the corresponding variations across calendar years are unclear 15. Summarily, although few studies analyzed the burden of leukemia from different aspects, and the latest disease burden of leukemia is still unknown. Therefore, in this study, we investigated temporal changes and group differences in the incidence and 5-year relative survival rate of all leukemia by sex, race, age, and subtype from 1975 to 2017 via the population-based Surveillance, Epidemiology, and End Results (SEER) database. Our results were aimed at facilitating evidence‐based assessment of the effectiveness of current prevention and treatment strategies on leukemia, as well as evaluating future leukemia burden and subsequent allocation of limited medical resources.

## Materials and methods

### Data source

We obtained incidence and survival rates of leukemia from the United States SEER database of the National Cancer Institute accessed on May 2020, via SEER*Stat software version 8.3.6.1 (https://seer.cancer.gov/). Three SEER research data with 9 Registries (SEER 9, 1975-2017), 13 Registries (SEER 13, 1992-2017), and 18 Registries (SEER 18, 2000-2017) were all obtained. The SEER 9, SEER 13, and SEER 18 databases cover about 9.4%, 13.4%, and 27.8% of the United States population based on the 2010 census 16.

For estimating the overall burden of leukemia, we aimed at enrolling all eligible leukemia patients with the following inclusion criteria: 1) the patients were diagnosed with leukemia using the Site recode ICD-O-3/WHO 2008 variable in the SEER database; 2) the age of patients were known; 3) the diagnosis year was from 1975 to 2017; 4) no limitation in age, subtype, and primary site. Considering the accuracy of disease diagnosis, the exclusion criterion was the diagnosed source as death certificate only and autopsy only The total number of leukemia patients during 1975-2017 with data in the SEER database was 480 818, of whom 140 171 were diagnosed during 1975-2017 (SEER 9), 133 904 were diagnosed during 1992-2017 (SEER 13), and 206 743 were diagnosed during 2000-2017 (SEER 18).

The demographic and clinical data, including the year of diagnosis, sex, race, age group, subtype, age-adjusted incidence rate, and 5-year relative rate, were used to assess the temporal trend of leukemia over the calendar year. According to the SEER database, the racial group was defined as white, black, other (American Indian/AK Native, Asian/Pacific Islander), and unknown population. The data on the unknown racial population were excluded when calculating the heterogeneity among races. The leukemia was categorized into 9 subtypes, namely, ALL, chronic lymphocytic leukemia (CLL), other lymphocytic leukemia, AML, acute monocytic leukemia (AMoL), chronic myeloid leukemia (CML), other myeloid/monocytic leukemia, other acute leukemia, and aleukemic, subleukemic and NOS based on the Site recode ICD-O-3/WHO 2008 in SEER database.

### Data analysis

The age-adjusted incidence rates were calculated using the 2000 United States Standard Population, and reported per 100 000 persons each year. For the 5-year survival analysis, the leukemia patients were further restricted cases with full follow-up records and the year of diagnosis as of 2012. The initial number of leukemia patients for follow-up less than 5 were further excluded. SEER*Stat was applied to calculate the average annual percent changes (APC) and corresponding *P* values of incidence and survival trends from the SEER databases and to conduct joinpoint regression analyses (Joinpoint Regression Program version 4.8.0.1, National Cancer Institute) 17. When analyzing the survival trend of different subtypes of leukemia over different ages at diagnosis, some related groups were combined because the sample size of some groups was too infrequent to be evaluated. All graphs were performed using the R program (Version 3.6.2), and the temporal trend over years was fitted and drawn using Adaptive Loess Regression. A two-sided *P* value of less than 0.05 was considered statistically significant.

### Ethical considerations

Ethics approval was exempted by the Ethics Committee of Qilu Hospital of Shandong University because the SEER is a publicly available database and all patient data were anonymous.

## Results

### Overall prevalence

According to the SEER 9 database, 140 171 patients developed leukemia from 1975 to 2017, including 80 475 males (57.4%) and 59 696 females (42.6%). Of these, white patients [120 724 (86.1%)] comprised the majority, followed by black patients [10 361 (7.4 %)], other racial patients [8186 (5.8 %)], and unknown racial patients [900 (0.6 %)]. The CLL [50 850 (36.3%)] and AML patients [38 199 (27.3%)] were the top 2 leading subtypes of all leukemia patients. More than half of the leukemia patients were diagnosed at 60 years old or older. The overall distributions of leukemia patients by sex, race, subtype, and age in the SEER 13 and the SEER 18 databases were similar to the SEER 9 (shown in Table 1).

### The secular trend of incidence of leukemia

**Figure 1:**
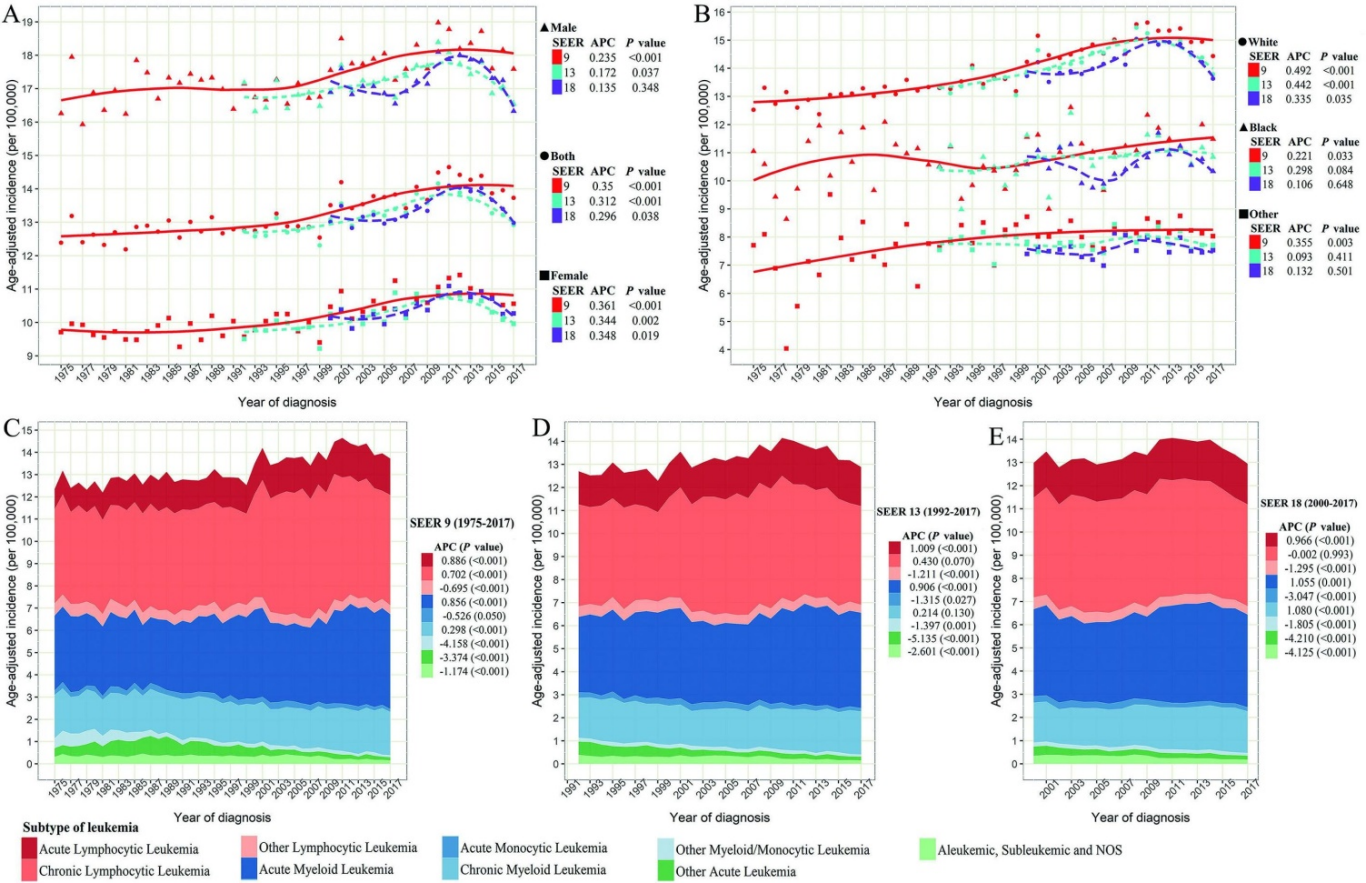
** The age-adjusted incidence of leukemia over years. (A)** The incidence of leukemia stratified by sex and SEER registry. **(B)** The incidence of leukemia stratified by race and SEER registries. **(C)** The incidence of leukemia by histological subtype in SEER 9 registry database. **(D)** The incidence of leukemia by histological subtype in SEER 13 registry database. **(E)** The incidence of leukemia by histological subtype in SEER 18 registry database. SEER: Surveillance, Epidemiology and End Results; APC: annual percentage change.

Figure 1 depicts the age-adjusted incidence of leukemia by sex, race, and leukemia subtype at each year in three SEER registry databases. SEER 18 and SEER 13 data, with a larger sample size of the United States and more recent calendar year, showed a slightly lower incidence of leukemia by sex and race than SEER 9 data. Although the difference in the incidence of leukemia in three SEER databases was observed, the temporal trend was somewhat parallel since 2000 in three SEER databases. The overall age-adjusted incidence of leukemia increased initially with 12.39 (95%CI: 11.85, 12.95) per 100 000 persons in 1975, reaching a peak in 2011 with 14.65 (95%CI: 14.22, 15.09) per 100 000 persons, and then gradually decreased with 13.73 (95%CI: 13.33, 14.13) per 100 000 persons in 2017 in SEER 9 (Table 1), with a steady increase (APC=0.350, *P* < 0.001) during this period. Notably, the incidence of leukemia peaked around 2011 in both men and women, and a decline of the incidence of leukemia in the recent 7 years could be observed, especially SEER 18 and SEER 13 (Figure 1A).

Figure 1A shows that men are more likely to diagnose leukemia than women, with a ratio of about 1.7:1. But the annual growth rate in leukemia is slightly higher more than that of men during the past 40 years. Concerning racial groups, the age-adjusted incidence of leukemia in the white population was the highest among all racial groups during the same period (white *vs* black *vs* others =13.99 *vs* 10.92 *vs* 7.99/100 000 person-years on average in SEER 9). White leukemia patients showed an increased incidence from 1975 to 2011, and then a decrease was observed in the recent 7 years. Among black and other racial populations, the incidence of leukemia remained relatively stable since 1992 (Figure 1B). Regarding the subtype of leukemia (Figure 1C-E), the steady upward trend in incidences of ALL and AML in three SEER databases could be found with all APCs more than 0.850 (*P* < 0.001). The incidence of CLL significantly increased from 1975 to around 2000, and then remained at a stable level until now. Although the incidence of CML dropped slightly around 2000, the incidence has been increasing in the past 20 years. In contrast, the incidences of other lymphocytic leukemia, AMoL, other myeloid/monocytic leukemia, other acute leukemia, and aleukemic, subleukemic and NOS were steadily declining over the past four decades in these three SEER databases.

**Figure 2:**
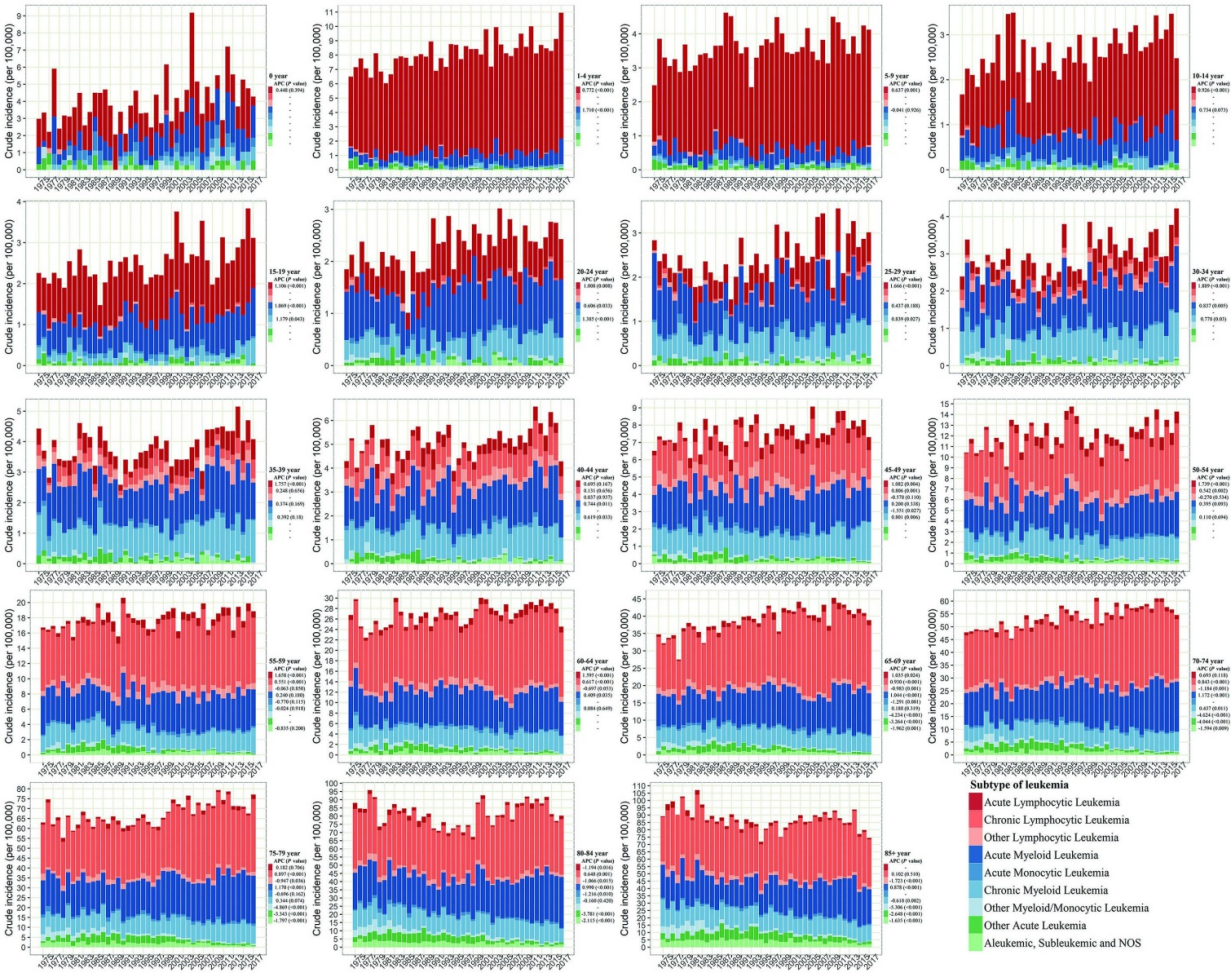
The annual incidence and proportion of all leukemia subtypes stratified by age groups in SEER 9 registry database, 1975-2017. SEER: Surveillance, Epidemiology and End Results; APC: annual percentage change.

**Figure 3:**
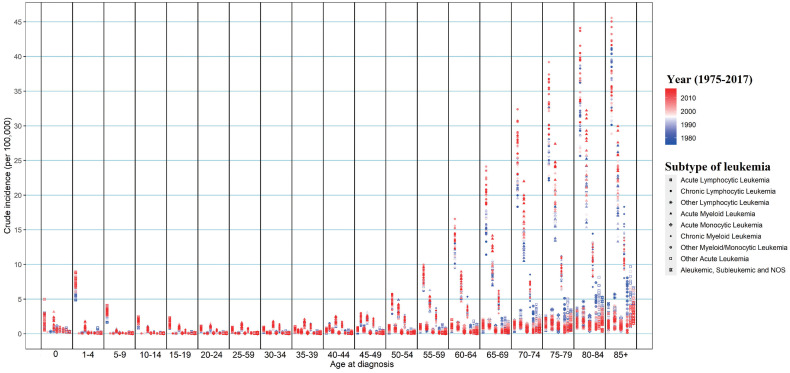
The change in incidences of all leukemia subtypes from 1975 to 2017 in SEER 9 registry database, stratified by age groups. SEER: Surveillance, Epidemiology and End Results.

We further assess the change of subtypes of leukemia across age groups in SEER 9 (Figure 2). ALL and AML were the leading subtypes in newborns under 1-year-old. ALL was the predominant subtype in under-15 children, and then with age increasing, AML gradually replaced its proportion, followed by CML and CLL. The incidence of ALL presented a significant growth trend among almost all people under-70 from 1975 to 2017, and the largest growth could be observed in people aged 30-34 with an APC of 1.889 (*P*<0.001). We could observe the incidence of AML increased among people aged 1-4, 15-24, 30-34, 40-44, and over 60 during the same period, and the incidence of AML remained unchanged among other age groups. Regarding CML, more and more young people aged 15-49 were diagnosed over the past four decades, and among them, the incidence of CML in people aged 20-24 increased the fastest, with an APC of 1.385 (*P*<0.001). But among the elderly over 85, the incidence of CML had declined, with an APC of -0.618 (*P*=0.002). Among the population aged 45-84, we could find the incidence of CLL was increasing gradually, especially in people aged 65-69 (APC=0.930, *P* < 0.001). The incidences of other lymphocytic leukemia, AMoL, other myeloid/monocytic leukemia, other acute leukemia, and aleukemic, subleukemic and NOS usually declined in the population over 65 from 1975 to 2017. Moreover, the overall change pattern of leukemia by subtype and age group was illustrated in Figure 3, which indicated the dominant subtypes of leukemia in the corresponding age group increased in the past 40 years and the incidence of all leukemia subtypes was relatively low in the population aged 20-39.

### Five-year survival trends of leukemia

**Figure 4:**
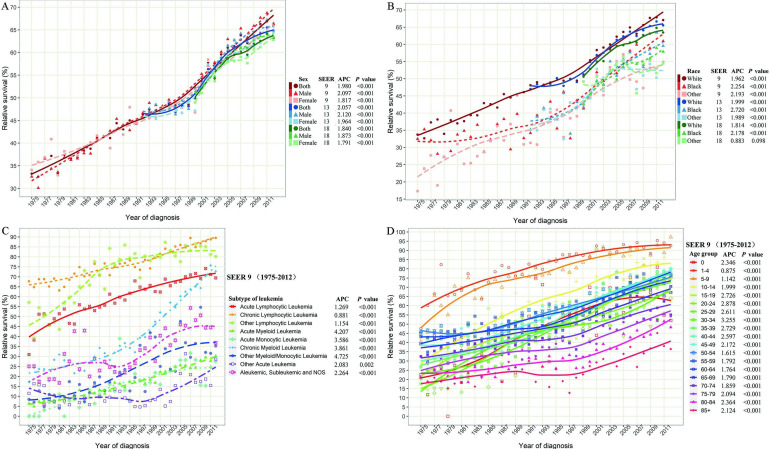
** The long-term survival of leukemia patients. (A)** The 5-year relative survival rate of leukemia patients' stratified by sex and SEER registry. **(B)** The 5-year relative survival rate of leukemia patients' stratified by race and SEER registry. **(C)** The 5-year relative survival rate of leukemia patients' stratified by histological subtype in SEER 9 registry database. **(D)** The 5-year relative survival rate of leukemia patients' stratified by age groups in SEER 9 registry database. SEER: Surveillance, Epidemiology and End Results; APC: annual percentage change.

In 1975, the 5-year relative survival rate of all leukemia patients was only 33.2% (Table 1). The 5-year survival rate of leukemia patients increased to 66.1% in 2012 in SEER 9 data, with an APC of 1.980 (*P*<0.001) from 1975 to 2012 (Figure 4A). Similar 5-year survival rates and temporal trends of leukemia patients were observed in three SEER databases, regardless of sex, throughout the monitoring period. However, we found that the 5-year survival rate of white leukemia patients in 2012 was the highest among all races (white *vs* black *vs* others =67.1% *vs* 60.9% *vs* 54.9% in SEER 9), and the racial difference in the 5-year survival rate of leukemia was continuously observed during the entire monitoring period (Figure 4B).

Concerning the subtype of leukemia, the 5-year survival rates of all subtypes increased from 1975 to 2012 (Figure 4C). Overall, the 5-year survival rates of all lymphocytic leukemia patients presented a better prognosis than other subtypes over the past 38 years. Compared with the survival rate in 1975, the rates for ALL, CLL, and other lymphocytic leukemia, increased by 124.5%, 32.1%, and 40.3% over the past decades, reaching 69.5%, 89.5%, and 80.2%, respectively, in 2012. In addition, the 5-year relative survival rates of CML increased dramatically from 17.2% in 1975 to 72.8% in 2012, with an APC of 3.861 (*P*<0.001). Although all APCs of AML, AMoL, and other myeloid/monocytic were more than 3.5, the recent 5-year relative survival rates of corresponding subtypes were still less than 35%.

Although the relative 5-year survival rates of leukemia in all age groups improved significantly in SEER 9 data (all *P*<0.001), the prognosis difference of leukemia patients was closely related to the age of onset (Figure 4D). The 5-year survival rate of leukemia patients aged 1-9 reached around 90% in 2012 and with the increase in age of diagnosis, the survival rate gradually declined. In particular, when the age of onset exceeded 70 years, the decline in survival rate was more obvious, and the relative 5-year survival rates of leukemia patients over 85 years old was only 36.5% in 2012.

**Figure 5:**
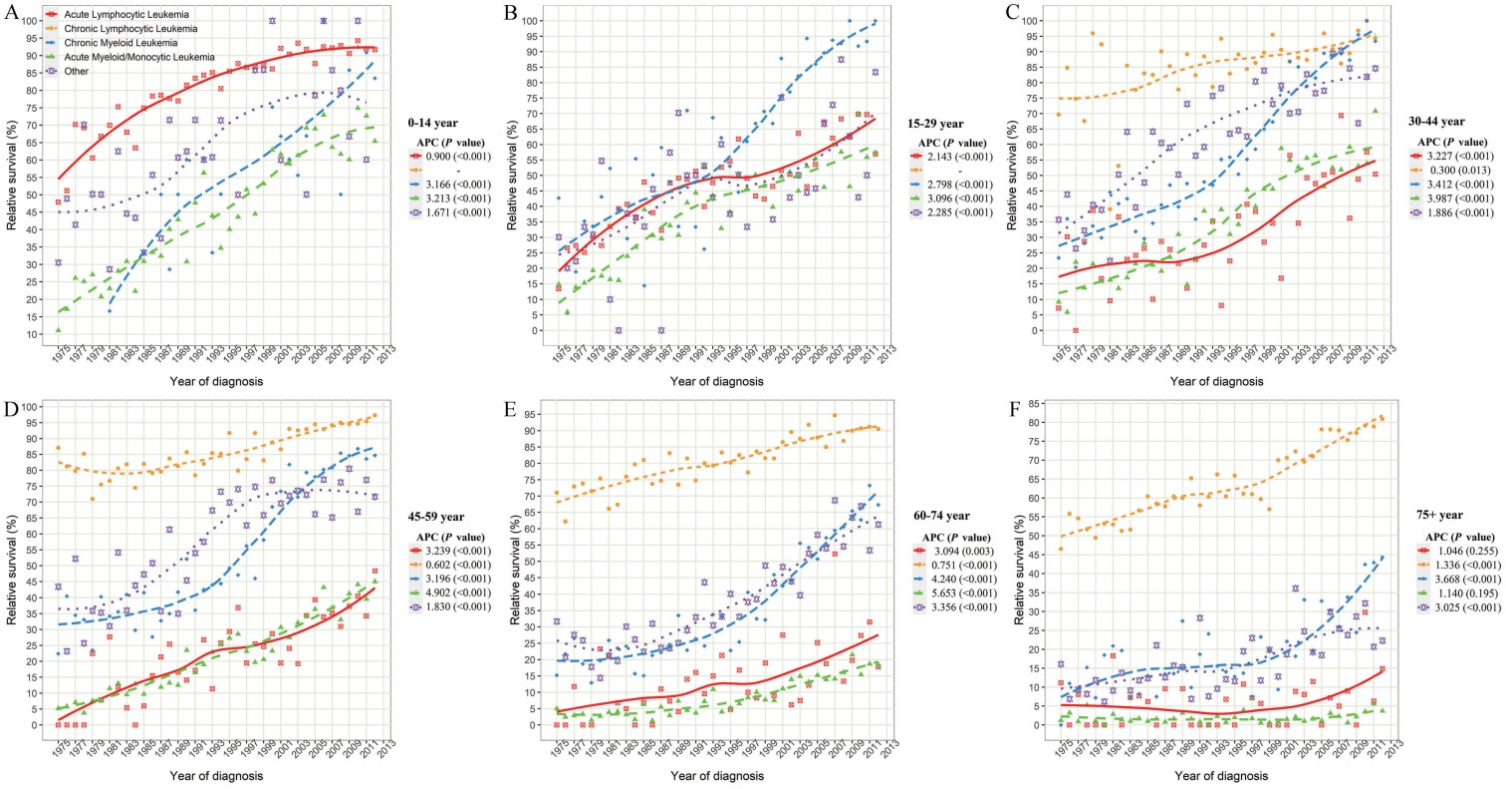
The 5-year relative survival rate of leukemia subtypes stratified by combined age groups in SEER 9 registry database, 1975-2017. SEER: Surveillance, Epidemiology and End Results; APC: annual percentage change.

**Figure 6:**
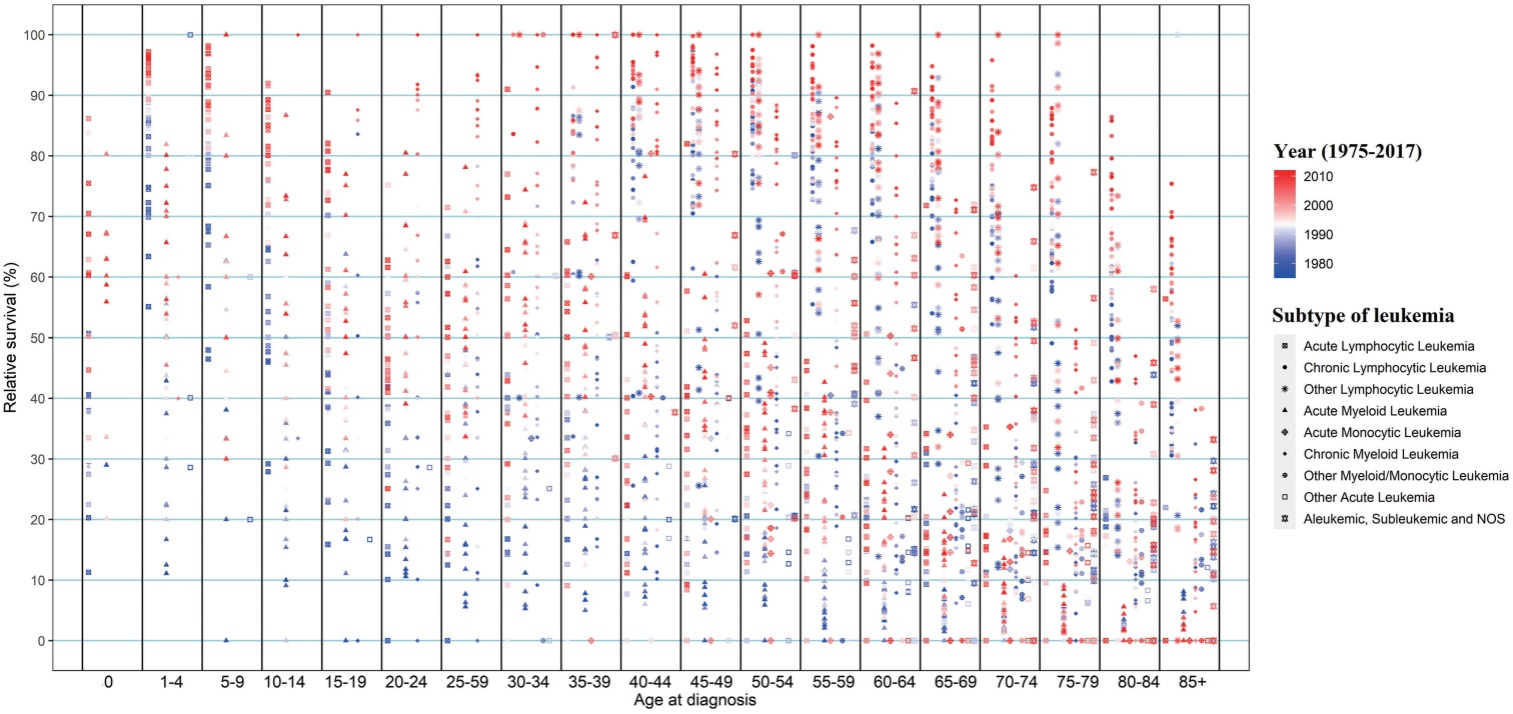
The change in 5-year relative survival of all leukemia subtypes from 1975 to 2012 in SEER 9 registry database, stratified by age groups. SEER: Surveillance, Epidemiology and End Results.

Considering the huge heterogeneity of subtypes of leukemia in different age groups, we further estimated the survival rate changes of leukemia subtypes across combined age groups (Figure 5). Except for CLL, the survival rates of other leukemia subtypes decreased at different degrees with the increase in age of diagnosis. In particular, the 5-year survival rates of ALL patients aged 0-14, 15-29, 30-44, 45-59, 60-74 and over 75 years were 91.7%, 57.0%, 50.5%, 48.4%, 17.8%, and 14.9%, respectively, in 2012. Meanwhile, with the increase of age, the survival rate of AML/AmoL patients also decreased markedly, especially the relative survival rate of AML/AmoL patients over 75 years old had been less than 5% in the past few decades (APC=1.140, *P*=0.195). In addition, the overall change pattern of the survival rate of leukemia patients by subtype and age group was illustrated in Figure 6, which indicated the prognosis of all young leukemia patients improved significantly over years, and only CLL and CML patients over 65 years old steadily increased in recent years.

## Discussion

In this large national population-based analysis of all leukemia patients in the United States, we found the overall incidence of leukemia was steadily increasing until 2011 from 12.39/100 000 in 1975 to 14.65/100 000 in 2011, and then the incidence of leukemia began to decline only in recent years (13.73/100 000 in 2017). Moreover, the long-term survival of leukemia patients significantly improved from 33.2% to 66.1% in 2012 from 1975 to 2012. However, we also noticed the huge disparities in the disease incidence and prognosis among different sexes, races, histological subtypes, and age groups.

The fluctuation in total leukemia incidence was observed, of which the exact reason was unclear. The reason for the continued increase in leukemia incidence rate before 2011 may be related to various factors, including raising awareness, improving diagnostic sensitivity, better reporting and recording new cases, and more risk factors exposure such as obesity, which has been associated with multiple leukemia subtype 18-20. Over the past half-century, innovations in leukemia treatment contribute to the steady increase in the 5-year survival rate in this study, and are heightened through national health policies and cancer control programs 21.

In the current study, the incidence of leukemia in males is around 1.7 times that of females, and the susceptibility of leukemia to men is also observed worldwide 22, 23. However, females had comparatively higher APC than males for age-adjusted leukemia incidence during the entire monitoring period. The potential reasons for the sex difference in leukemia incidence maybe originate from female reproductive factors, exogenous hormones, and lifestyles, affecting potentially immune function and the carcinogenesis process 24-26. However, we observed that the 5-year survival rate of all leukemia patients did not differ in sex throughout the monitoring period.

In terms of racial differences, the incidence of leukemia among white people is consistently higher than that of black people and other people over the past four decades in the three SEER registration systems. Conversely, the survival prognosis of white patients was always better than that of black patients and other racial patients. This racial heterogeneity is well documented, which could be interpreted by the socioeconomic, cultural, biological, and genetic differences 10, 21, 27, 28.

We also note a trend disparity in the incidence of different leukemia subtypes. The incidences of ALL and AML in most age groups were significantly increased over the past four decades. The incidence of CLL significantly increased from 1975 to around 2000, and remained stable since then. Although the incidence of CML was relatively low among people under 50 years old, the corresponding incidence was increasing in the past 20 years. In contrast, the incidences of all other subtypes, including other lymphocytic leukemia, AMoL, other myeloid/monocytic leukemia, other acute leukemia, and aleukemic, subleukemic and NOS, showed a continued decline over the past four decades, especially among people above 65 years old. The increase in the incidence of ALL, AML, CLL, and CML may partly stem from the increase in their incidence, and partly due to the reclassification of other leukemia subtypes.

The incremental advancement in leukemia prognosis has been achieved in almost all histological subtypes and age groups over time, except that the survival rate of ALL/AML/AmoL patients over 75 years old remained virtually unchanged. The progress in laboratory and clinical research on leukemia has produced many new concepts and treatments in past decades, which will undoubtedly continue to improve the prognosis of leukemia 2, 29. Freireich et. al. summarized the half-century of evolutions in outcomes and treatments for leukemia subtypes at the 50th anniversary of the American Society of Clinical Oncology 30. We observed that even for the same leukemia subtype, the survival rate would gradually decrease with the increase in age of diagnosis, which is somewhat consistent with prior studies that demonstrated worsening survival among the older population, compared to the younger population 11, 21, 27, 31. In particular, among above-75 leukemia patients other than CLL the 5-year relative survival rate is almost below 45% in 2012, which partly may be explained as the patients with other comorbidities, prior organ dysfunction, and unbearable for intensive chemotherapy, lack of insurance 32-35. As a result, new therapeutic strategies, such as monoclonal antibodies, CAR-T cells, better supportive care, more effective disease monitoring, and combinations with other investigational agents, need be developed and verified to improve the prognosis of all leukemia types, especially in the elder patients 7, 20, 32.

In interpreting our results, some limitations need to be considered. Firstly, the lack of information on individual treatments and other clinic parameters such as leukocyte count, comorbidities, and personal lifestyles. However, our epidemiological descriptive study comprehensively provides the most updated, real-world information on the incidence and prognosis of leukemia by sex, race, age, and subtype with the largest population over the past four decades from the national population-based registry system. Secondly, due to the complexity and heterogeneity of hematological malignancies, the corresponding coding has undergone many changes over time, which may affect the trends of certain subtypes (as described above). Moreover, a more detailed leukemia histological subtype of leukemia based on the WHO classification is not estimated, because case numbers of certain subtypes of leukemia in some of the age-specific groups are too small to stably detect changes in morbidity and survival rates 23. Finally, possible external validation researches could provide an important comparison for our results, but the large sample size in the current study somewhat allay concerns for the reliability of our results.

In conclusion, our study provides a comprehensive overview of leukemia over the past 43 years in the United States. The SEER data shows the overall incidence of leukemia has been steadily increasing until 2011, and then declines in recent years. In particular, the main subtypes of leukemia, including ALL, AML, CLL, and CML, present an incremental increase. On the contrary, the incidences of all other subtypes are gradually declined over the past four decades. Moreover, the long-term survival of leukemia patients significantly improved over time in almost all subgroups. However, the huge disparities in the leukemia incidence and prognosis among different sexes, races, histological subtypes, and age groups, indicate that the potential causes of control and innovative treatments need to be explored to reduce the incidence and improve the prognosis in certain specific populations.

## Figures and Tables

**Table 1:** The characteristics of leukemia patients in three SEER Databases

Variables	SEER 9	SEER 13	SEER 18
**Sex**			
Both	140 171 (100.0%)	133 904 (100.0%)	206 743 (100.0%)
Men	80 475 (57.4%)	77 282 (57.7%)	119 245 (57.7%)
Women	59 696 (42.6%)	56 622 (42.3%)	87 498 (42.3%)
**Race**			
White	120 724 (86.1%)	112 090 (83.7%)	175 017 (84.7%)
Black	10 361 (7.4%)	10 149 (7.6%)	16 806 (8.1%)
Other	81 86 (5.8%)	10 402 (7.8%)	12 198 (5.9%)
unknown	900 (0.6%)	1263 (0.9%)	2722 (1.3%)
**Diagnosis era**			
1975-1983	20 473 (14.61%)	0 (0%)	0 (0%)
1984-1991	21 354 (15.23%)	0 (0%)	0 (0%)
1992-1995	11 949 (8.52%)	16 655 (12.44%)	0 (0%)
1995-1999	12 592 (8.98%)	17 489 (13.06%)	0 (0%)
2000-2005	21 640 (15.44%)	29 350 (21.92%)	60 328 (29.18%)
2006-2011	24 490 (17.47%)	33 529 (25.04%)	68 719 (33.24%)
2012-2017	27 673 (19.74%)	36 881 (27.54%)	77 696 (37.58%)
**Household income (USD)**			
<45000	4736 (3.38%)	4674 (3.49%)	18 189 (8.8%)
45000-55000	12 257 (8.74%)	11 767 (8.79%)	30 444 (14.73%)
55000-65000	22 987 (16.4%)	45 804 (34.21%)	58 739 (28.41%)
65000-75000	21 893 (15.62%)	25 947 (19.38%)	37 754 (18.26%)
≥75000	41 879 (29.88%)	45 576 (34.04%)	61 483 (29.74%)
Unknown	36 419 (25.98%)	136 (0.1%)	134 (0.06%)
**Location**			
Metropolitan	87 401 (62.35%)	117 961 (88.09%)	181 593 (87.84%)
Nonmetropolitan	15 296 (10.91%)	14 712 (10.99%)	24 861 (12.03%)
Unknown	37 474 (26.73%)	1231 (0.92%)	289 (0.14%)
**Phenotype**			
B-cell/pre-B/B-precursor	35 065 (25.02%)	47 832 (35.72%)	88 317 (42.72%)
T-cell	1065 (0.76%)	734 (0.55%)	1066 (0.52%)
Other	2137 (1.52%)	2753 (2.06%)	4183 (2.02%)
Unspecified	101 904 (72.7%)	82 585 (61.67%)	113 177 (54.74%)
**Subtype**			
***Lymphocytic Leukemia***	70 969 (50.6%)	68 338 (51.0%)	105 892 (51.2%)
Acute Lymphocytic Leukemia	15 201 (10.8%)	16 709 (12.5%)	25 374 (12.3%)
Chronic Lymphocytic Leukemia	50 850 (36.3%)	47 223 (35.3%)	74 133 (35.9%)
Other Lymphocytic Leukemia	4918 (3.5%)	4406 (3.3%)	6385 (3.1%)
***Myeloid and Monocytic Leukemia***	61 714 (44.0%)	59 857 (44.7%)	92 654 (44.8%)
Acute Myeloid Leukemia	38 199 (27.3%)	38 399 (28.7%)	59 648 (28.9%)
Acute Monocytic Leukemia	2397 (1.7%)	2311 (1.7%)	3685 (1.8%)
Chronic Myeloid Leukemia	18 843 (13.4%)	17 747 (13.3%)	26 995 (13.1%)
Other Myeloid/Monocytic Leukemia	2275 (1.6%)	1400 (1.0%)	2326 (1.1%)
***Other Leukemia***	7488 (5.3%)	5709 (4.3%)	8197 (4.0%)
Other Acute Leukemia	4169 (3.0%)	2916 (2.2%)	3703 (1.8%)
Aleukemic, Subleukemic and NOS	3319 (2.4%)	2793 (2.1%)	4494 (2.2%)
**Age group**			
0 year	638 (0.5%)	651 (0.5%)	966 (0.5%)
1-4 year	5019 (3.6%)	5221 (3.9%)	7552 (3.7%)
5-9 year	2787 (2%)	2918 (2.2%)	4330 (2.1%)
10-14 year	2048 (1.5%)	2231 (1.7%)	3442 (1.7%)
15-19 year	2006 (1.4%)	2163 (1.6%)	3379 (1.6%)
20-24 year	1842 (1.3%)	1918 (1.4%)	2890 (1.4%)
25-29 year	2095 (1.5%)	2080 (1.6%)	2983 (1.4%)
30-34 year	2520 (1.8%)	2550 (1.9%)	3630 (1.8%)
35-39 year	3117 (2.2%)	3062 (2.3%)	4445 (2.2%)
40-44 year	3896 (2.8%)	3998 (3.0%)	5962 (2.9%)
45-49 year	5396 (3.8%)	5560 (4.2%)	8685 (4.2%)
50-54 year	7896 (5.6%)	7766 (5.8%)	12 265 (5.9%)
55-59 year	10 403 (7.4%)	9859 (7.4%)	16 009 (7.7%)
60-64 year	13 234 (9.4%)	12 089 (9.0%)	19 513 (9.4%)
65-69 year	15 806 (11.3%)	14 345 (10.7%)	22 527 (10.9%)
70-74 year	17 240 (12.3%)	15 596 (11.6%)	23 340 (11.3%)
75-79 year	16 644 (11.9%)	15 556 (11.6%)	23 796 (11.5%)
80-84 year	14 073 (10.0%)	13 278 (9.9%)	20 694 (10.0%)
85+ year	13 511 (9.6%)	13 063 (9.8%)	20 335 (9.8%)
**Survival status**			
Alive	40 770 (29.09%)	51 542 (38.49%)	92 300 (44.64%)
Dead	99 401 (70.91%)	82 362 (61.51%)	114 443 (55.36%)
**Overall age-adjusted incidence (per 100,000)**			
Baseline (1975/1992/2000)	12.39 (11.85, 12.95)	12.74 (12.34, 13.14)	13.01 (12.75, 13.27)
2017	13.73 (13.33, 14.13)	12.92 (12.59, 13.25)	12.98 (12.75, 13.21)
**Overall 5-year relative survival (%)**			
Baseline (1975/1992/2000)	33.2 (30.9, 35.6)	45.4 (43.6, 47.3)	50.1 (48.9, 51.3)
2012	66.1 (64.2, 68.0)	64.7 (63.1, 66.3)	63.1 (62.0, 64.3)

SEER: Surveillance, Epidemiology and End Results.

## Abbreviations

APC: annual percent changes
SEER: Surveillance, Epidemiology, and End Results
ALL: acute lymphoblastic leukemia
AML: acute myeloid leukemia
CLL: chronic lymphocytic leukemia
AMoL: acute monocytic leukemia
CML: chronic myeloid leukemia
